# Acceptance of the COVID-19 Vaccine Among Postpartum Women in Five Countries

**DOI:** 10.1089/whr.2024.0202

**Published:** 2025-04-28

**Authors:** Eun Young Lee, Li-Yin Chien, Yan-Shing Chang, Seo Ah Hong, Kelly Pereira Coca

**Affiliations:** ^1^ Department of Nursing, Catholic Kkottongnae University, Cheongju, Republic of Korea.; ^2^ Institute of Community Health Care, College of Nursing, National Yang Ming Chiao Tung University, Taipei, Taiwan.; ^3^ Florence Nightingale Faculty of Nursing, Midwifery & Palliative Care, King’s College London, London, United Kingdom.; ^4^ Department of Public Health Administration, Faculty of Public Health, Mahidol University, Bangkok, Thailand.; ^5^ Women’s Health Nursing Department, Escola Paulista de Enfermagem, Universidade Federal de São Paulo, São Paulo, Brazil.

**Keywords:** COVID-19, vaccine, attitudes and beliefs, acceptance, postpartum

## Abstract

**Objectives:**

This study identified factors associated with COVID-19 vaccine acceptance and willingness to receive the vaccine among postpartum women in the United Kingdom, Thailand, Taiwan, Brazil, and South Korea.

**Methods:**

An online cross-sectional survey was designed and conducted from July to November 2021. Data from 3,253 women who were up to 6 months postpartum in the United Kingdom, Thailand, Taiwan, Brazil, and South Korea were analyzed. Simple and multiple logistic regression analyses were performed.

**Results:**

Approximately 72% of women had received at least one dose of the vaccine with a range from 97.0% in Brazil to 25.2% in South Korea. Among five countries, positive attitudes and beliefs toward the COVID-19 vaccine were associated with COVID-19 vaccine acceptance (odds ratio [OR]: 1.41; 95% confidence interval [CI]: 1.35, 1.47). Higher education, paid employee status, and urban residence were associated with COVID-19 vaccine acceptance. The country-specific analysis results showed that attitudes and beliefs toward the COVID-19 vaccine were a strong predictors of COVID-19 vaccine acceptance and willingness to receive it among postpartum women.

**Conclusion:**

Positive attitudes and beliefs toward the COVID-19 vaccine and better COVID-19-related knowledge, attitudes, and practices are important for acceptance of the vaccine in postpartum women. Therefore, there is a need for sufficient and clear communication on the novel vaccine’s safety and efficacy to improve awareness and attitudes among postpartum women.

## Introduction 

Since the World Health Organization (WHO) declared the COVID-19 pandemic in March 2020, there have been nearly 776 million confirmed cases and over 7 million deaths worldwide as of November 2024.^1^ Vaccination is a key measure to mitigate the impact of the COVID-19 pandemic on individuals and communities.^2^ After reporting the effectiveness of vaccinations against COVID-19 in December 2020,^3,4^ mass vaccination began immediately. However, information on vaccine safety and efficacy during pregnancy and postpartum was limited because pregnant and postpartum women were excluded from phase three clinical trials of COVID-19 vaccines.^5^ Although the WHO recommended vaccination in pregnant and postpartum women as the benefits of vaccination outweighed the potential risks, ambiguous guidance on vaccination and the safety concerns of the COVID-19 vaccines driven by rapid vaccine development, a lack of knowledge about the mRNA-based vaccines, and insufficient information of the long-term effects made some of these women hesitant to receive vaccines.^6–10^

Postpartum women are a population vulnerable to COVID-19 due to ongoing physiological changes after childbirth. Women infected with COVID-19 in this period have more severe symptoms than the general population.^11^ COVID-19 vaccination is recommended for postpartum women to prevent COVID-19 infection and minimize the severity of infection along with potential benefits including passive immunity through breast milk.^12^

The WHO’s behavioral and social drivers of the vaccination framework highlights the domain of “what people are thinking and feeling,” which includes disease risk appraisal and vaccine confidence.^13,14^ Thus, knowledge, attitudes, and practices (KAP) related to COVID-19 played a major role in disease risk appraisal and adherence to infection control measures such as vaccination.^15,16^ Positive attitudes and beliefs regarding the COVID-19 vaccine are an important predictor of vaccine acceptance.^8,9,13,17^ Moreover, a longitudinal study reported that attitudes and beliefs regarding the COVID-19 vaccine have a persisting influence on future decision-making processes to obtain a COVID-19 vaccine.^18^ However, there are few studies on attitudes and beliefs regarding the COVID-19 vaccine related to breastfeeding, which is a major concern of postpartum women.

During the pandemic, the United Kingdom and Brazil, which experienced higher COVID-19 incidence and death rates, commenced vaccination for pregnant and postpartum women in December 2020^19^ and in April 2021,^20^ respectively. However, South Korea and Taiwan, which have lower rates of COVID-19 incidence and death, started vaccinating later than those countries.^21,22^ COVID-19 vaccination in South Korea was offered in February 2021 and vaccination for pregnant and postpartum women in October 2021.^23^ These different contexts and experiences in each country may have influenced the levels of COVID-19-related KAP, attitudes and beliefs regarding the COVID-19 vaccine, and vaccine acceptance in postpartum women, which means these factors needed to be addressed regarding COVID-19 as well as the next global health crisis.

In addition, studies have reported that having a child, younger age, lower education, unemployment, low socioeconomic status, no history of COVID-19 positivity, and little concern about COVID-19 were associated with low vaccine acceptance in postpartum women.^17,24–27^ Although a few studies have reported through cross-country surveys conducted early in the pandemic,^17,28^ there are fewer studies on COVID-19 vaccine acceptance and associated factors among postpartum women using the same survey across countries when mass vaccinations began in 2021.^8,9^ Therefore, this study aimed to identify the factors associated with COVID-19 vaccine acceptance and the willingness to receive it among postpartum women in the United Kingdom, Thailand, Taiwan, Brazil, and South Korea.

## Methods

### Study design and data collection

We carried out a cross-sectional, self-administered, and anonymous web-based survey using Google Forms from July to November 2021, during mass vaccinations against COVID-19, in five countries: the United Kingdom, Thailand, Taiwan, Brazil, and South Korea. We included women who (1) were up to 6 months postpartum, (2) were aged 18–49 years old (20–49 years for Taiwan), and (3) understood the written language of their resident participating country. We excluded women who (1) have the youngest baby over 7 months of age, (2) were not living in one of the participating countries, and (3) could not read the questions. Of the 3,507 responses, a total of 3,253 eligible responses (858 in the United Kingdom, 840 in Thailand, 614 in Taiwan, 560 in Brazil, and 381 in South Korea) were included in the study analyses, excluding 254 responses that did not meet inclusion criteria or had incomplete responses.

We developed a structured questionnaire in English and then translated it into Thai, Chinese, Portuguese, and Korean. Each local language questionnaire was backward-translated into English to assess the quality of the translation. The content validity of the questionnaire was assessed by experts and postpartum women in each country. The pilot test was conducted by 3–10 postpartum women from each participating country. Unclear, misleading, or sensitive questions were revised to improve clarity. The first part of the final questionnaire included an online informed consent form, the second part consisted of COVID-19-related questions, the third part was COVID-19 vaccine-related questions, and the last part included sociodemographic questions. We invited survey participation *via* emails, personal and health care professional networks, and social media platforms (WhatsApp, Twitter, Facebook, and Instagram).

### Ethics statement

We reported participant recruitment and data collection proceeded after receiving approval from the ethics committees of the five universities: King’s College London, United Kingdom (HR/DP-20/21-22651, RESCM-20/21-22651), Mahidol University, Thailand (No. 2021/03-042), National Yang Ming Chiao Tung University, Taiwan (No. YM110060E), the Federal University of São Paulo, Brazil (No. 4.858.900), and Catholic Kkottongnae University, South Korea (No. 2-7008080-A-N-01-202103-HR-003). We conducted the study following the relevant institutional guidelines from the Declaration of Helsinki. We provided the study objectives and assured confidentiality to each participant at the introduction of the survey. All participants voluntarily signed an online informed consent form before answering the survey questions.

## Measures

### Dependent variables

We measured COVID-19 vaccine acceptance with the question, “Have you received a COVID-19 vaccine?” (options were Yes, No, or Don’t know). In the analyses, respondents who answered “No” or “Don’t know” were considered as no receipt, whereas those answering “Yes” were considered as vaccine receivers. We asked about the willingness to receive a vaccine based on the questions, “If a COVID-19 vaccine was made available to you this week, would you definitely get it?” (options were Yes, No, Don’t know, or I have already received a COVID-19 vaccine). We categorized respondents with “Yes” into “those willing to receive,” whereas those with “No” or “Don’t know” were “those not willing to receive.” Willingness to receive a COVID-19 vaccine was calculated among those who had not received one.

### Independent variables

We developed questions on attitudes and beliefs toward the COVID-19 vaccine related to breastfeeding based on a previous study by the Imperial College London’s Institute of Global Health Innovation.^29^ These items included (1) “A COVID-19 vaccine is safe for breastfeeding women and babies,” (2) “Breastfeeding women are not allowed to receive a COVID-19 vaccine,” (3) “Women should stop breastfeeding in order to receive a COVID-19 vaccine,” and (4) “I am worried about potential side effects of a COVID-19 vaccine.” They were answered based on a five-point Likert-type scale (1 = strongly disagree to 5 = strongly agree). We reverse-coded and summed Questions 2, 3 and 4. A total score ranged from 4 to 20 with a higher score indicating more positive attitudes and beliefs toward the COVID-19 vaccine. Cronbach’s alpha was 0.75. The internal consistency of attitudes and beliefs toward the COVID-19 vaccine was acceptable in this study.

We drew and modified from previous studies^30,31^ and WHO’s COVID-19 infection prevention and control measures^32^ to develop questions regarding COVID-19-related KAP. COVID-19-related knowledge included nine questions; for example, “COVID-19 cannot spread through respiratory droplets of infected individuals” (options were True, False, or Don’t know). We awarded one point to an appropriate answer, and the total score ranged from 0 to 9 with a higher score suggesting better knowledge. Cronbach’s alpha for the nine questions was 0.76. We measured attitudes toward the severity and prevention of COVID-19 using seven questions (*e.g.,* “COVID-19 is preventable”) answered on a five-point Likert-type scale (1 = strongly disagree to 5 = strongly agree). The total scores ranged from 7 to 35. A higher score indicated a more positive attitude. Cronbach’s alpha for these seven questions was 0.87. We assessed COVID-19-related precaution practices using six questions such as, “During the last seven days, did you avoid touching the eyes, nose and mouth with unwashed hands?” answered using a four-point Likert-type scale (1 = never, 2 = occasionally, 3 = sometimes, and 4 = always). The total score ranged from 6 to 24 with a higher score showing a more adequate practice. Cronbach’s alpha for the six questions was 0.80.

We collected information about parity (one child/two or more children), maternal age (18–29 years/30–39 years/40–49 years), education (high school or below/college or above), employment (yes/on maternity leave/housemaker or unemployed), marital status (married/single, divorced, widowed, or separated), residence (urban/rural), worrying about COVID-19 (yes/no), and ever diagnosis as COVID-19 positive (yes/no). We investigated the change in food security status before and during the COVID-19 pandemic with two questions: “Did you ever run out of food before the end of the month or cut down on the amount you ate to feed others in 2019 before COVID-19?” and “Did you ever run out of food before the end of the month or cut down on the amount you ate to feed others during COVID-19 in 2020–2021?” A change in food security was categorized as (1) insecure before and during COVID-19, (2) secure to insecure, (3) insecure to secure, and (4) secure.

### Statistical analysis

We conducted all analyses using SAS 9.3 (SAS Institute Inc., Cary, NC, USA). We calculated and presented frequency and percentage for categorical variables and mean and standard deviation for continuous variables. We used the Chi-square test or analysis of variance (ANOVA) to compare variables among five countries. We carried out simple and multiple logistic regressions to estimate the crude and adjusted odds ratio (COR and AOR, respectively), with a 95% confidence interval (CI), to explore the associated factors. We entered independent variables with *p* value <0.05 in bivariate analyses into multiple logistic regression. Statistical significance was considered with *p* value <0.05.

## Results

**FIG. 1.**
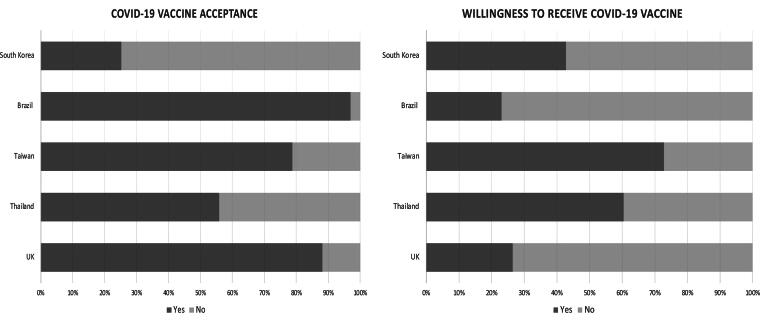
COVID-19 vaccine acceptance and willingness to receive a COVID-19 vaccine in participants from five countries.

Of the 3,253 total women participating in the survey, the majority were 30–39 years old (61.6%), on maternity leave (59.2%), had a college or higher education level (75.8%), lived in an urban area (72.6%) and were married (95.5%) (Table 1). Approximately 72% of women received at least one dose of the vaccine ranging from 25.2% in South Korea to 97.0% in Brazil. The percentage of willingness to receive the COVID-19 vaccine was 52.3% overall among women who did not receive the vaccine ranging from 23.1% in Brazil to 72.9% in Taiwan (Fig. 1).

**Table 1.** Characteristics of Postpartum Women and COVID-19-Related Knowledge, Attitudes, and Practices by Countries

Variables	Total (*n* = 3253)	United Kingdom (*n* = 858)	Thailand (*n* = 840)	Taiwan (*n* = 614)	Brazil (*n* = 560)	South Korea (*n* = 381)	*p* value
	*n* (%)/(Mean ± SD)	*n* (%)/(Mean ± SD)	*n* (%)/(Mean ± SD)	*n* (%)/(Mean ± SD)	*n* (%)/(Mean ± SD)	*n* (%)/(Mean ± SD)	
Parity							
One child	1853 (57.0)	393 (45.8)	436 (51.9)	424 (69.1)	393 (70.2)	207 (54.6)	<0.001
Two or more children	1398 (43.0)	465 (54.2)	404 (48.1)	190 (30.9)	167 (29.8)	172 (45.4)	
Maternal age							
18–29 years	1094 (33.6)	186 (21.7)	489 (58.2)	204 (33.2)	164 (29.3)	51 (13.4)	<0.001
30–39 years	2005 (61.6)	619 (72.1)	318 (37.9)	397 (64.7)	360 (64.3)	311 (81.6)	
40–49 years	154 (4.7)	53 (6.2)	33 (3.9)	13 (2.1)	36 (6.4)	19 (5)	
Education							
High school or below	787 (24.2)	175 (20.4)	458 (54.5)	35 (5.7)	85 (15.2)	34 (8.9)	<0.001
College or above	2465 (75.8)	682 (79.6)	382 (45.5)	579 (94.3)	475 (84.8)	347 (91.1)	
Employment							
Yes	564 (17.3)	31 (3.6)	357 (42.5)	28 (4.6)	87 (15.6)	61 (6.0)	<0.001
On maternity leave	1926 (59.2)	777 (90.6)	171 (20.4)	487 (79.3)	368 (65.8)	123 (32.3)	
Housemaker/unemployed	762 (23.4)	50 (5.8)	312 (37.1)	99 (16.1)	104 (18.6)	197 (51.7)	
Marital status							
Married	3105 (95.5)	840 (97.9)	759 (90.4)	605 (98.5)	521 (93.0)	380 (99.7)	<0.001
Single/divorced/widowed/separated	148 (4.6)	18 (2.1)	81 (9.6)	9 (1.4)	39 (7.0)	1 (0.3)	
Residence							
Urban	2360 (72.6)	541 (63.1)	382 (45.5)	535 (87.1)	542 (97.1)	360 (94.5)	<0.001
Rural	891 (27.4)	317 (37.0)	458 (54.5)	79 (12.9)	16 (2.9)	21 (5.5)	
Food insecurity during COVID-19 pandemic							
No change (insecure–insecure)	298 (9.2)	22 (2.6)	236 (28.1)	—	19 (3.4)	21 (5.5)	<0.001
Worse (secure–insecure)	340 (10.5)	72 (8.4)	180 (21.4)	—	75 (13.5)	13 (3.4)	
Better (insecure–secure)	27 (0.8)	4 (0.5)	13 (1.6)	—	5 (0.9)	5 (1.3)	
No change (secure–secure)	2584 (79.5)	760 (88.6)	411 (48.9)	614 (100.0)	457 (82.2)	342 (89.8)	
Worrying about COVID-19							
No	2252 (69.3)	666 (77.6)	498 (59.3)	466 (75.9)	361 (64.9)	261 (68.5)	<0.001
Yes	997 (30.7)	192 (22.4)	342 (40.7)	148 (24.1)	195 (35.1)	120 (31.5)	
Ever diagnosed as COVID-19 positive							
No	2836 (87.2)	731 (85.2)	697 (83.0)	613 (99.8)	420 (75.0)	375 (98.4)	<0.001
Yes	417 (12.8)	127 (14.8)	143 (17.0)	1 (0.2)	140 (25.0)	6 (1.6)	
COVID-19-related knowledge (0–9)	7.8 (±1.7)	8.5 (±0.9)	6.7 (±2.1)	8.6 (±0.9)	7.7 (±1.4)	7.4 (±2.1)	<0.001
COVID-19-related attitudes (7–35)	29.8 (±4.5)	30.3 (±3.8)	28.5 (±5.3)	31.6 (±2.6)	30.5 (±4.6)	27.6 (±4.9)	<0.001
COVID-19-related practices (6–24)	20.5 (±3.6)	19.5 (±3.4)	19.6 (±4.3)	21.7 (±2.8)	21.6 (±2.8)	20.9 (±3.2)	<0.001

COVID-19, coronavirus disease 2019; *n*, number; SD, standard deviation.

Table 1 shows COVID-19-related KAP in each country. The total score of COVID-19-related knowledge was 7.8 ± 1.7 ranging from 6.7 ± 2.1 in Thailand to 8.6 ± 0.9 in Taiwan. The total scores of COVID-19-related attitudes were higher in Taiwan (31.6 ± 2.6), Brazil (30.5 ± 4.6), and the United Kingdom (30.3 ± 3.8) than in Thailand and South Korea (28.5 ± 5.3 and 27.6 ± 4.9, respectively). Lastly, the total score of COVID-19-related practices was 20.5 ± 3.6 ranging from 19.5 ± 3.4 in the United Kingdom to 21.7 ± 2.8 in Taiwan.

Table 2 shows responses to each question about attitudes and beliefs toward the COVID-19 vaccine. The total score of attitudes and beliefs regarding the COVID-19 vaccine was lower in countries in Asia (South Korea: 11.01 ± 2.69, Thailand: 13.32 ± 2.74, and Taiwan: 14.12 ± 2.45) than in the United Kingdom and Brazil (16.98 ± 2.77 and 17.41 ± 2.65, respectively). For example, regarding the question “A COVID-19 vaccine is safe for breastfeeding women and babies” (Question 1), mothers who answered “agree or strongly agree” were lower in Asian countries (South Korea: 34.2%, Thailand: 55.8%, and Taiwan 78.2%) than Brazil and the United Kingdom (both over 80%). Meanwhile, responses to “agree or strongly agree” to the questions, “Breastfeeding women are not allowed to take a COVID-19 vaccine” (Question 2) and “Women should stop breastfeeding in order to take a COVID-19 vaccine” (Question 3), were greater in South Korea (30.7% and 38.3%, respectively) and Thailand (16.6% and 14.7%, respectively) compared to the other countries (7.3% and 0.9% for Brazil, 2.6% and 8.3% for Taiwan and 1.5% and 0.8% for the United Kingdom). Responses “agree to strongly agree” to the question, “I am worried about potential side effects of a COVID-19 vaccine” (Question 4), were greater in Asian countries (Taiwan: 83.1%, South Korea: 73.8%, and Thailand: 56.8%) than the United Kingdom and Brazil (39.4% and 20.2%, respectively).

**Table 2.** Comparing of Attitudes and Beliefs Toward the COVID-19 Vaccine by Countries

Items	Total	United Kingdom	Thailand	Taiwan	Brazil	South Korea	*p* value
1. A COVID-19 vaccine is safe for breastfeeding women and babies							
Strongly disagree (%)	3.1	2.2	5.1	0.5	1.6	6.8	<0.001
Disagree (%)	5.7	2.6	8.1	1.1	1.4	21.3	
Undecided (%)	20.6	11.4	31.0	20.2	8.0	37.8	
Agree (%)	32.2	28.8	43.1	30.6	27.9	24.7	
Strongly agree (%)	38.4	55.0	12.7	47.6	61.1	9.5	
2. Breastfeeding women are not allowed to take a COVID-19 vaccine							
Strongly disagree (%)	46.9	83.5	26.2	28.7	67.9	8.7	<0.001
Disagree (%)	25.2	7.9	24.9	53.8	18.8	28.1	
Undecided (%)	17.9	7.1	32.4	15.0	6.1	32.6	
Agree (%)	7.0	0.6	13.5	1.6	2.9	22.3	
Strongly agree (%)	3.0	0.9	3.1	1.0	4.5	8.4	
3. Women should stop breastfeeding in order to receive a COVID-19 vaccine							
Strongly disagree (%)	49.0	88.3	26.7	24.3	78.4	6.0	<0.001
Disagree (%)	23.2	4.6	29.6	46.1	17.7	22.6	
Undecided (%)	17.6	6.4	29.1	21.3	3.0	33.1	
Agree (%)	7.5	0.4	11.8	6.4	0.4	26.5	
Strongly agree (%)	2.7	0.4	2.9	2.0	0.5	11.8	
4. I am worried about potential side effects of a COVID-19 vaccine							
Strongly disagree (%)	16.0	23.1	7.9	2.3	42.0	1.6	<0.001
Disagree (%)	14.4	20.1	12.6	7.0	23.2	4.5	
Undecided (%)	16.8	17.5	22.7	7.7	14.6	20.2	
Agree (%)	35.0	26.0	43.9	51.0	13.6	41.5	
Strongly agree (%)	17.8	13.4	12.9	32.1	6.6	32.3	
Total score (Mean ± SD)	14.87 ± 3.45	16.98 ± 2.77	13.32 ± 2.74	14.12 ± 2.45	17.41 ± 2.65	11.01 ± 2.69	<0.001

COVID-19, coronavirus disease 2019; SD, standard deviation.

Selected variables associated with COVID-19 vaccine acceptance (*p* < 0.05 from bivariate analyses) were employed in the multivariable model (Table 3). A high school or lower education level (AOR: 0.71, 95% CI: 0.55, 0.93) compared with college or above, employment (AOR: 1.79, 95% CI: 1.34, 2.38 for working women and AOR: 1.62, 95% CI: 1.21, 2.17 for women being on paid maternity leave) compared with housemaker/unemployed, urban residence (AOR: 1.33, 95% CI: 1.06, 1.67) compared with rural residence, and a one-unit score increase in attitudes and beliefs regarding the COVID-19 vaccine (AOR: 1.41; 95% CI: 1.35, 1.47) were associated with vaccine acceptance. Regarding the willingness to vaccinate (Table 3), worrying about COVID-19, attitudes and beliefs regarding the COVID-19 vaccine, and COVID-19-related KAP were employed in the multiple logistic regression models. Worrying about COVID-19 (AOR: 1.39, 95% CI: 1.01, 1.92 compared to no worry), a one-unit increase in attitudes and beliefs regarding the COVID-19 vaccine (AOR: 1.37, 95% CI: 1.28, 1.47), in COVID-19-related knowledge (AOR: 1.15, 95% CI: 1.06, 1.26), and in the COVID-19-related practice (AOR: 1.09, 95% CI: 1.04, 1.14) were associated with willingness to receive the COVID-19 vaccine.

**Table 3.** Factors Associated with COVID-19 Vaccine Acceptance and Willingness to Receive the Vaccine Among Postpartum Women

Variables	COVID-19 vaccine acceptance (*n* = 3,253)		Willingness to receive a COVID-19 vaccine (*n* = 899)	
	COR (95% CI)	AOR (95% CI)	COR (95% CI)	AOR (95% CI)
Country (ref. South Korea)				
United Kingdom	22.00 (16.14, 30.01)	5.20 (3.42, 7.91)	0.48 (0.29, 0.79)	0.22 (0.12, 0.40)
Thailand	3.75 (2.87, 4.91)	2.86 (1.98, 4.12)	2.05 (1.50, 2.81)	1.68 (1.16, 2.45)
Taiwan	11.05 (8.18, 14.94)	4.95 (3.44, 7.12)	3.59 (2.28, 5.65)	1.80 (1.08, 3.01)
Brazil	94.83 (55.52, 161.96)	20.52 (11.28, 37.34)	0.40 (0.11, 1.49)	0.34 (0.08, 1.54)
Parity (ref. Two or more children)				
One child	1.25 (1.07, 1.46)	1.23 (1.00, 1.51)	1.07 (0.83, 1.40)	
Maternal age (ref. 40–49 years)				
18–29 years	0.66 (0.45, 0.97)	0.93 (0.56, 1.55)	1.34 (0.69, 2.59)	
30–39 years	1.03 (0.71, 1.50)	1.05 (0.64, 1.71)	1.04 (0.54, 2.01)	
Education (ref. college or above)				
High school or below	0.45 (0.38, 0.53)	0.71 (0.55, 0.93)	1.05 (0.80, 1.39)	
Employment (ref. Housemaker/unemployed)				
Yes	2.04 (1.63, 2.56)	1.79 (1.34, 2.38)	1.14 (0.80, 1.63)	
On paid maternity leave	5.46 (4.48, 6.65)	1.62 (1.21, 2.17)	0.92 (0.67, 1.28)	
On unpaid maternity leave	2.56 (1.94, 3.37)	1.35 (0.94, 1.93)	1.24 (0.79, 1.95)	
Marital status (ref. Single/divorced/widowed/separated)				
Married	1.22 (0.86, 1.74)		0.67 (0.36, 1.22)	
Residence (ref. Rural)				
Urban	1.53 (1.29, 1.80)	1.33 (1.06, 1.67)	0.96 (0.73, 1.27)	
Food insecurity during COVID-19 pandemic				
No change (insecure–insecure)	0.41 (0.32, 0.52)	1.26 (0.91, 1.75)	1.38 (0.95, 2.02)	
Worse (secure–insecure)	0.53 (0.42, 0.67)	0.79 (0.57, 1.10)	1.47 (1.00, 2.16)	
Better (insecure–secure)	0.41 (0.19, 0.87)	0.92 (0.35, 2.41)	2.04 (0.61, 6.84)	
Worrying about COVID-19 (ref. No)				
Yes	0.81 (0.68, 0.95)	0.98 (0.79, 1.21)	1.69 (1.27, 2.23)	1.39 (1.01, 1.92)
Ever diagnosed as COVID-19 positive (ref. No)				
Yes	1.09 (0.86, 1.37)		1.09 (0.73, 1.63)	
Attitudes and beliefs toward the COVID-19 vaccine	1.57 (1.52, 1.62)	1.41 (1.35, 1.47)	1.31 (1.24, 1.39)	1.37 (1.28, 1.47)
COVID-19-related knowledge	1.26 (1.20, 1.31)	0.96 (0.90, 1.02)	1.17 (1.09, 1.25)	1.15 (1.06, 1.26)
COVID-19-related attitudes	1.09 (1.07, 1.11)	1.02 (1.00, 1.05)	1.09 (1.06, 1.13)	1.03 (1.00, 1.07)
COVID-19-related practices	1.04 (1.02, 1.06)	1.02 (0.99, 1.05)	1.12 (1.08, 1.17)	1.09 (1.04, 1.14)

COVID-19, coronavirus disease 2019; COR, crude odds ratio; AOR, adjusted odds ratio; CI, confidence interval; ref., reference.

Table 4 shows that attitudes and beliefs toward the COVID-19 vaccine were associated with COVID-19 vaccine acceptance and a willingness to receive the vaccine in each country. Additionally, COVID-19-related KAP was not associated with vaccine acceptance in Thailand and Taiwan, whereas a positive COVID-19-related attitude in the United Kingdom, COVID-19-related practice in Brazil, and COVID-19-related knowledge in South Korea, respectively, were associated with vaccine acceptance.

**Table 4.** Factors Associated with COVID-19 Vaccine Acceptance and Willingness to Receive a COVID-19 Vaccine Among Postpartum Women by Countries

Variables	COVID-19 vaccine acceptance		Willingness to receive a COVID-19 vaccine^a^	
	COR (95% CI)	AOR (95% CI)	COR (95% CI)	AOR (95% CI)
United Kingdom				
Attitudes and beliefs toward the COVID-19 vaccine	1.79 (1.63, 1.98)	1.82 (1.62, 2.05)	2.09 (1.52, 2.87)	4.07 (1.89, 8.77)
COVID-19-related knowledge	1.37 (1.13, 1.66)	1.10 (0.82, 1.48)	1.60 (0.91, 2.82)	0.79 (0.34, 1.87)
COVID-19-related attitudes	1.23 (1.17, 1.29)	1.15 (1.06, 1.25)	1.31 (1.13, 1.51)	1.58 (1.12, 2.23)
COVID-19-related practices	1.10 (1.04, 1.17)	0.94 (0.86, 1.04)	1.18 (1.03, 1.35)	0.93 (0.68, 1.26)
Thailand				
Attitudes and beliefs toward the COVID-19 vaccine	1.30 (1.23, 1.38)	1.28 (1.20, 1.37)	1.36 (1.22, 1.51)	1.51 (1.32, 1.74)
COVID-19-related knowledge	1.09 (1.02, 1.16)	0.94 (0.87, 1.02)	1.27 (1.13, 1.41)	1.15 (1.01, 1.12)
COVID-19-related attitudes	1.00 (0.97, 1.02)	0.99 (0.96, 1.02)	1.05 (1.01, 1.10)	1.06 (1.01, 1.12)
COVID-19-related practices	1.00 (0.97, 1.03)	0.99 (0.96, 1.03)	1.12 (1.06, 1.18)	1.11 (1.04, 1.19)
Taiwan				
Attitudes and beliefs toward the COVID-19 vaccine	1.48 (1.35, 1.63)	1.51 (1.37, 1.66)	1.40 (1.16, 1.68)	1.52 (1.22, 1.89)
COVID-19-related knowledge	1.06 (0.85, 1.31)	0.88 (0.69, 1.12)	1.48 (0.98, 2.24)	1.86 (1.10, 3.14)
COVID-19-related attitudes	1.00 (0.93, 1.08)	0.97 (0.89, 1.06)	1.01 (0.88, 1.16)	0.95 (0.80, 1.13)
COVID-19-related practices	1.01 (0.95, 1.08)	0.99 (0.92, 1.07)	1.00 (0.89, 1.13)	0.92 (0.78, 1.08)
Brazil				
Attitudes and beliefs toward the COVID-19 vaccine	1.60 (1.35, 1.90)	1.61 (1.30, 2.00)	—	—
COVID-19-related knowledge	1.05 (0.75, 1.46)	0.86 (0.53, 1.40)	—	—
COVID-19-related attitudes	1.13 (1.05, 1.20)	0.98 (0.86, 1.11)	—	—
COVID-19-related practices	1.30 (1.15, 1.46)	1.22 (1.00, 1.48)	—	—
South Korea				
Attitudes and beliefs toward the COVID-19 vaccine	1.17 (1.07, 1.28)	1.16 (1.06, 1.28)	1.19 (1.07, 1.31)	1.26 (1.12, 1.41)
COVID-19-related knowledge	1.09 (0.97, 1.23)	1.18 (1.01, 1.38)	1.20 (1.06, 1.36)	1.15 (0.98, 1.35)
COVID-19-related attitudes	1.03 (0.98, 1.08)	1.00 (0.94, 1.07)	1.05 (1.00, 1.10)	0.98 (0.92, 1.04)
COVID-19-related practices	1.05 (0.97, 1.14)	1.06 (0.96, 1.17)	1.15 (1.06, 1.25)	1.13 (1.01, 1.25)

Adjusted for parity, maternal age, education, employment, marital status, residence, food insecurity during COVID-19 pandemic, worry about COVID-19, and ever diagnosed as COVID-19 positive.

^a^ Brazil was excluded due to low number of respondents.

COVID-19, coronavirus disease 2019; COR, crude odds ratio; AOR, adjusted odds ratio; CI, confidence interval; ref., reference.

## Discussion

The current study demonstrated factors associated with COVID-19 vaccine acceptance and willingness to receive the vaccine among 3,253 postpartum women from the United Kingdom, Thailand, Taiwan, Brazil, and South Korea using the same survey questionnaire during the mass vaccination period.

A higher proportion of women in Brazil and the United Kingdom had received at least one dose of COVID-19 vaccine than those in Asian countries during the survey period. Although the total score of COVID-19-related KAP was high in Taiwan, the total score of attitudes and beliefs toward the COVID-19 vaccine was higher in Brazil and the United Kingdom than the three Asian countries.

Before this survey, incidence and death rates of COVID-19 were higher in the United Kingdom and Brazil than in South Korea, Taiwan, and Thailand.^33^ Therefore, mass vaccination in the United Kingdom began earlier than in South Korea and Taiwan. The COVID-19 vaccination rate in the United Kingdom was over 60% in August 2021, whereas the vaccination rate in South Korea was less than 20% in August and later increased to 80% at the end of November 2021.^21^ Meanwhile, South Korea and Taiwan, which had low incidence and death rates of COVID-19, continued to take a nonpharmacological approach (*e.g.,* health education, wearing a face mask, social distancing, stringent quarantine measures, *etc.*) and closely monitored the short-term consequences of novel vaccines.^21,22^ Perceptions of the higher risk of COVID-19 infection and the negative consequences of COVID-19 infection along with mass vaccination by the government have influenced attitudes and beliefs regarding the COVID-19 vaccine and might have contributed to higher vaccine acceptance in Brazil and the United Kingdom.^34,35^

Consistent with previous studies,^17,36^ positive attitudes and beliefs toward the COVID-19 vaccine were a significant predictor of COVID-19 vaccine acceptance in postpartum women in five countries. Although the level of attitudes and beliefs regarding the COVID-19 vaccine varied depending on the context of each country, this study confirmed that attitudes and beliefs regarding the COVID-19 vaccine related to breastfeeding are an important factor in predicting COVID-19 vaccine acceptance in postpartum women.

Concern about the adverse effects and safety of COVID-19 vaccines were the most likely reason for vaccine hesitancy among mothers.^37,38^ To support positive attitudes and beliefs regarding the COVID-19 vaccines, public health agencies and health care professionals should provide women with sufficient scientific evidence on the efficacy and safety of novel vaccines.^23^ Thus, primary health care provides sufficient information about disease, vaccination, and breastfeeding to postpartum women and the government can improve public awareness through ongoing nationwide campaigns.

In the current study, education levels and employment status were significantly related to COVID-19 vaccine acceptance in postpartum women. Women who lived in urban areas were more likely to be vaccinated. COVID-19-related morbidity and mortality were higher for more deprived groups such as immigrants and those with low-wage occupations, low-incomes, and a lower education level.^39^ Likewise, groups that were educated, employed, and urban dwelling mothers were more susceptible to COVID-19 infections and were exposed to more information on the benefits and risks of vaccination.^40^ Although they are under social pressure to get vaccinated to work or to live in densely populated cities,^41,42^ well-educated, salaried, urban-dwelling mothers are better situated to benefit from the health care system, which likely reflects differences in access and resources (*e.g.,* time, working from home).^43^ Therefore, various strategies need to be developed to increase the acceptance rate of the COVID-19 vaccine among postpartum women who have low education levels, are unemployed or housewives, and live in a rural area.^40^

This study presented that maternal age and parity were not significantly associated with COVID-19 vaccine acceptance. A systematic review of hospital-based observational studies^8^ reported that older age and multiparity were associated with COVID-19 vaccine acceptance in a few studies, however, most studies did not find a significant association, which agrees with our study’s findings.

Our study revealed that a willingness to receive a COVID-19 vaccine in postpartum women was related to worrying about COVID-19, attitudes and beliefs toward the COVID-19 vaccine, and COVID-19-related knowledge and practices. The findings are consistent with previous studies.^17,44^ A study that collected data from 17,871 pregnant women and mothers in 16 countries reported that confidence in vaccine safety or effectiveness, worrying about COVID-19, and compliance with mask guidelines were the strongest predictors of a willingness to receive COVID-19 vaccines.^17^

Our study added the association between COVID-19 knowledge and willingness to receive a COVID-19 vaccine. Education level was associated with COVID-19-related knowledge and practices^15^ that influence the willingness to accept COVID-19 vaccines. Therefore, health care professionals should support mothers’ decisions on COVID-19 vaccinations by providing accurate information about COVID-19 and accounting for the level of education of the mothers.

Although our findings provide important information to enhance vaccination in postpartum women, there are a few limitations. As data were collected through a cross-sectional online survey, generalizations should be made cautiously and causal relationships cannot be established. There may be a selection bias at work because study participants were limited to those who had access to the online survey. Furthermore, the voluntary nature of the survey might have contributed to that selection bias. Last, there is a risk of social desirability bias when using self-reported information. Because acknowledging vaccine receipt did not specify whether the postpartum mothers received the vaccine during pregnancy or after birth, cautions should be practiced when interpreting the results.

## Conclusion

This cross-national comparison study demonstrated that positive attitudes and beliefs regarding the COVID-19 vaccine were important factors in predicting COVID-19 vaccine acceptance and willingness to receive it among postpartum women in the United Kingdom, Thailand, Taiwan, Brazil, and South Korea during the mass vaccination period. In the analysis by country, better COVID-19-related KAP as well as attitudes and beliefs regarding the COVID-19 vaccine were important factors for the novel vaccine’s acceptance in postpartum women. These findings suggest that sufficient and clear communication about vaccine safety and efficacy is critical to improve attitudes and beliefs toward the COVID-19 vaccines in postpartum women. Public health agencies and health care professionals should improve awareness of novel vaccines among socioeconomically vulnerable women and develop strategies for vaccination campaigns that reflect the context of the country.

## Abbreviations Used

ANOVA: analysis of variance
AOR: adjusted odds ratio
CI: confidence interval
COR: crude odds ratio
COVID: Coronavirus disease 2019
KAP: knowledge, attitudes, and practices
SD: Standard Deviation
UK: United Kingdom
WHO: World Health Organization
